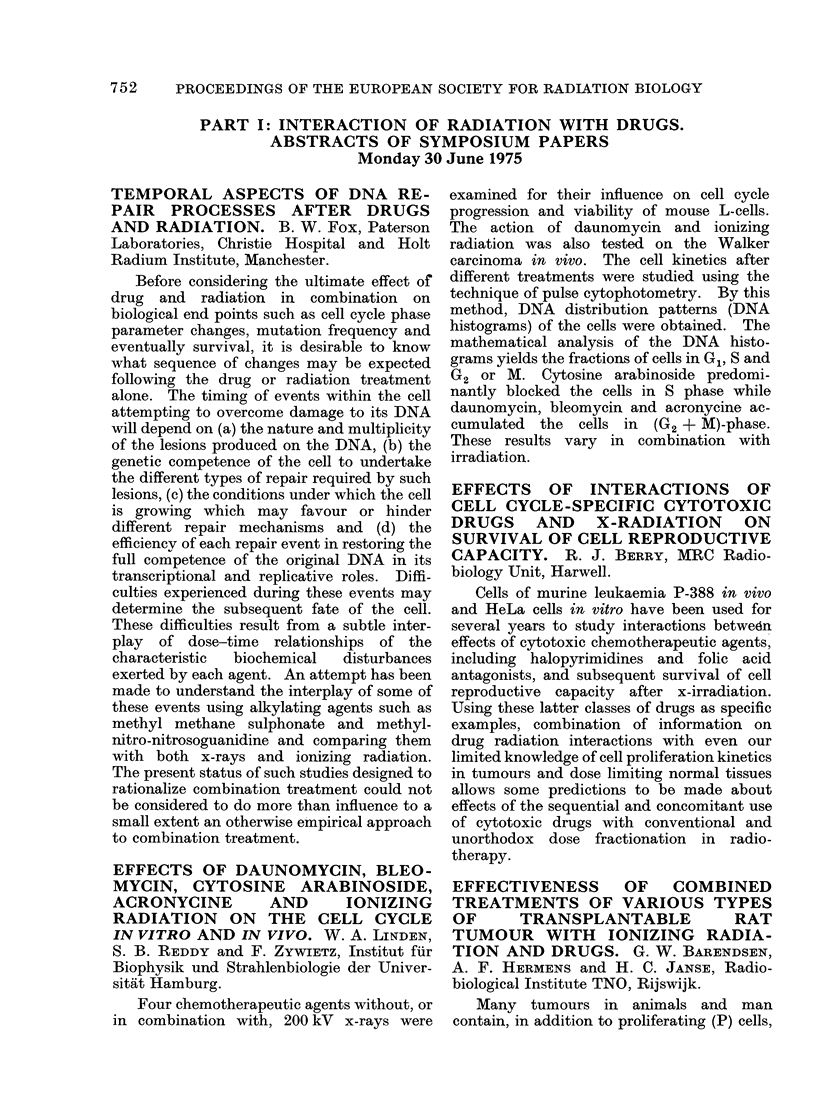# Proceedings: Effects of interactions of cell cycle-specific cytotoxic drugs and X-radiation on survival of cell reproductive capacity.

**DOI:** 10.1038/bjc.1975.294

**Published:** 1975-12

**Authors:** R. J. Berry


**EFFECTS OF INTERACTIONS OF CELL CYCLE-SPECIFIC CYTOTOXIC DRUGS AND X-RADIATION ON SURVIVAL OF CELL REPRODUCTIVE CAPACITY.** R. J. Berry, MRC Radiobiology Unit, Harwell.

Cells of murine leukaemia P-388 *in vivo* and HeLa cells *in vitro* have been used for several years to study interactions between effects of cytotoxic chemotherapeutic agents, including halopyrimidines and folic acid antagonists, and subsequent survival of cell reproductive capacity after x-irradiation. Using these latter classes of drugs as specific examples, combination of information on drug radiation interactions with even our limited knowledge of cell proliferation kinetics in tumours and dose limiting normal tissues allows some predictions to be made about effects of the sequential and concomitant use of cytotoxic drugs with conventional and unorthodox dose fractionation in radiotherapy.